# Patellofemoral Joint Replacement and Nickel Allergy: An Unusual Presentation

**DOI:** 10.1155/2015/635082

**Published:** 2015-10-12

**Authors:** Farhan Syed, Edward Jenner, Mohammad Faisal

**Affiliations:** Trauma & Orthopaedics, Warwick Hospital, Lakin Road, Warwick CV34 5BW, UK

## Abstract

Metal allergy is an unusual complication of joint replacement that may cause aseptic loosening and necessitate joint revision surgery. We present the case of nickel allergy causing aseptic loosening following patellofemoral joint replacement (PFJR) in a 54-year-old male. Joint revision surgery to a nickel-free total knee replacement was performed with good results. Our literature review shows that there is no evidence to guide the management of metal allergy in PFJR. The evidence from studies of total knee replacement is limited to retrospective case series and case reports and gives contradictory recommendations. The optimal management strategy for metal allergy in PFJR is not clear. We recommend allergy testing in patients with history of metal allergy and use of an allergen-free implant in those with positive tests. As there is no gold standard test to establish metal allergy, the choice of test should be guided by availability and recommendation from the local unit of dermatology and allergy testing. We recommend investigation for metal allergy in patients with implant loosening where other causes have been excluded.

## 1. Introduction

The role of metal allergy in failure following arthroplasty is poorly understood. In one of the earliest studies by Elves et al. looking at metal sensitivity in patients undergoing arthroplasty, it was found to be as high as 38% based on patch testing [1]. Although the study suggested a higher incidence of aseptic loosening in metal allergy group, it failed to establish a cause-effect relationship between metal sensitivity and failure of arthroplasty. We present a case of patellofemoral joint replacement failure in a patient who was later diagnosed with nickel sensitivity.

## 2. Case Reports

**Figure 1:**
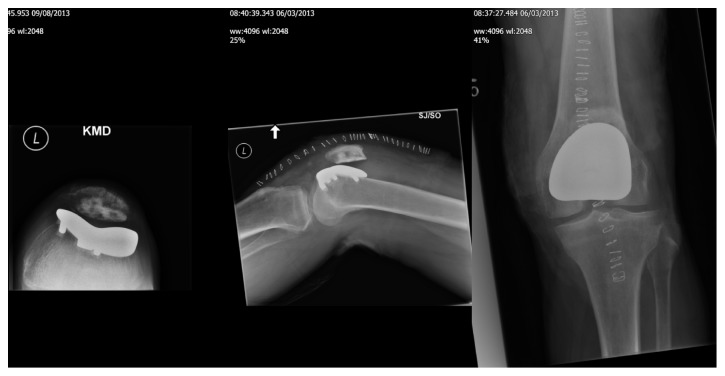
Postoperative radiograph of patellofemoral joint replacement.

A 54-year-old male patient was referred by the physiotherapist with 1 year history of anterior left knee pain. He had sustained a patellar tendon tear, requiring surgical repair 30 years ago. Clinical examination was mainly suggestive of patellofemoral arthritis. This was confirmed on plain radiographs and MRI. A knee arthroscopy was performed that showed grade 2 changes in the trochlea with grade 3 changes on the patella. At six-month review the patient reported no significant improvement in symptoms; he was therefore listed for patellofemoral joint replacement (PFJR). Following his surgery using the Zimmer Gender Solutions Patello-Femoral Joint his symptoms progressively worsened (Figure 1).

**Figure 2:**
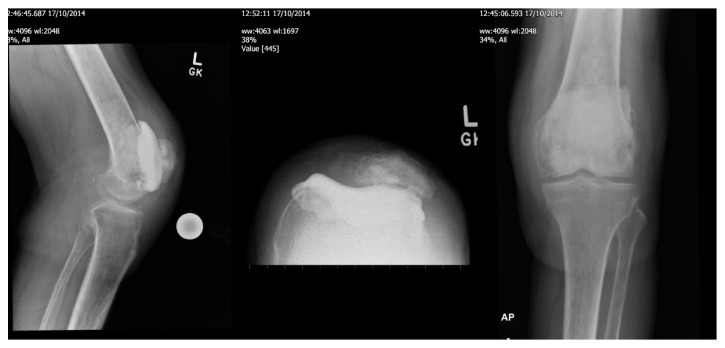
Postoperative radiograph of 1st stage revision with cement spacer, also seen is the cystic lesion on femoral side packed with bone graft.

The patient was systemically well; however, his mobility and left knee range of motion worsened to the extent that he required 2 crutches. His infection screen, including full blood count, CRP, and plasma viscosity, was not raised. All possible nonoperative measures were exhausted therefore we decided to perform a single stage revision to a total knee replacement. Intraoperatively, a large cyst was discovered under the femoral component. This necessitated a change of plan to a 2-stage revision. Multiple samples were taken and sent for microbiological and histological study. The cyst was curetted and packed with bone graft and a cement spacer was placed (Figure 2).

**Figure 3:**
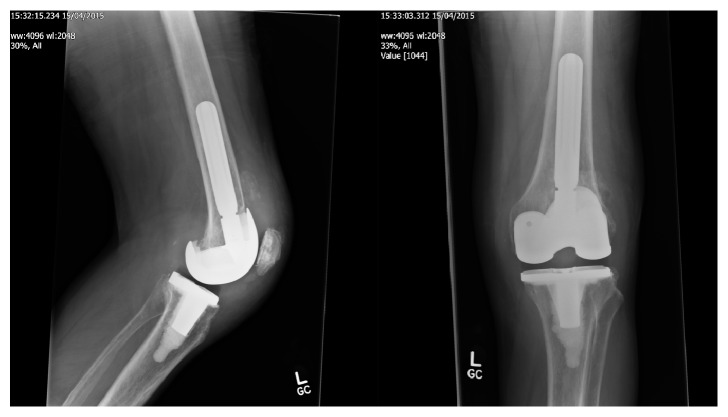
Postoperative radiograph of 2nd stage revision with nickel-free prosthesis.

The microbiological studies were negative and histopathological studies showed nonspecific inflammatory response. In the absence of any other cause of implant failure metal allergy was considered. The patient was referred to the allergy clinic for further investigation. A patch test was strongly positive for nickel allergy. This patient went on to have a 2nd stage revision using a nickel-free revision knee system (Figure 3), and at 6-month follow-up showed dramatic improvement in his symptoms.

## 3. Literature Review and Discussion

As there was no data about metal allergy specifically looking at patellofemoral joint replacement, our literature review, discussion, and conclusion were drawn from studies looking at metal allergy in total knee arthroplasty.

Granchi et al. in their systematic review of metal sensitivity in total joint replacement concluded that metal sensitivity testing might be useful in patients with failed joint replacements, especially those with metal on metal articulation [2]. Pinson et al. in their systematic review recommended hypersensitivity testing preoperatively in patients with reported history of metal sensitivity [3]. However, Thomas et al. in their original article with a sample size of 32 patients looked at correlation between patch test and aseptic loosening and interestingly failed to find any correlation [4].

A team of dermatologists looked at how patch testing can influence surgical practice with regard to metal implantation in orthopaedic patients [5]. The patch test was carried out in 31 preimplantation and 41 postimplantation subjects. 21 of the preimplantation group tested positive and all of them went on to receive allergen-free implant. With regard to the postimplantation group, six of ten patients who were positive to the metal in their implant showed resolution of symptoms following implant removal. Based on these findings they suggested preimplantation patch testing in patients with clinical history of metal sensitivity, whereas patch test positivity in postimplantation patients requires a case by case approach from the clinician.

Schalock and Thyssen, in their questionnaire responses from clinicians attending the European Society of Contact Dermatitis (ESCD) 2012 and the American Contact Dermatitis Society 2013 meetings, found patch testing as the best and most widely used test for metal hypersensitivity reaction [6].

Innocenti et al. in their case series looked at identifying patients with metal sensitivity in 1007 consecutive patients undergoing TKA [7]. Based on history only 2.6% were possibly metal sensitive. They developed a protocol combining history, patch testing, ELISA, LTT (lymphocyte transformation test), and conmicroscopy. Using this combination of investigations the actual metal sensitivity was 0.49%.

Niki et al. used modified lymphocyte stimulation test (mLST) to Ni, Co, Cr, and Fe to screen 92 patients undergoing primary knee arthroplasty [8]. 26% demonstrated sensitivity; however only 5 out of those patients demonstrated symptomatic metal sensitivity, and it showed strong correlation when it came to chromium metal. Based on their study the authors recommended routine preoperative screening, especially for chromium.

Anand et al. in their case report highlighted how metal sensitivity can mimic infection and if it has been excluded then metal sensitivity should be investigated and considered as a cause of patient symptoms [9]. Thomsen et al. presented a case report of eczematous reaction, pain, and decreased movement following a cobalt chromium knee prosthesis [10]. After ruling out infection, patient went on to have revision with geometrically similar prosthesis having antiallergic multicoating of ZrN, resulting in disappearance of symptoms and improved range of movement. However it is pertinent to note that metal allergy was a clinical diagnosis as the lymphocyte transformation test showed no increased value for any metal ions (i.e., cobalt, chromium, and nickel).

On the contrary, Thienpont and Berger published a case report of a patient having a metal sensitivity to cobalt, chromium, and nickel based on skin patch testing remaining asymptomatic with a cobalt and chromium prosthesis at 2-year follow-up [11].

Most of the evidence with regard to metal sensitivity in orthopaedic patients is in form of case series and case reports. Only a small subgroup of patients with clinical history and lab testing for metal allergy actually went on developing symptomatic metal allergy. With regard to investigations there are various tests available to test for metal allergy, with patch test being the most widely used test. A more comprehensive study is needed to establish a definite correlation between metal allergy and adverse symptoms following joint replacement.

## 4. Conclusion

Based on the limited evidence available we recommend that patients with clinical history of specific metal allergy should undergo allergy testing and preferably receive an allergen-free implant if found positive. We would limit investigations for metal allergy to those postimplantation patients in whom other obvious causes like infection and loosening have been excluded. The choice of test should be guided by availability and recommendation from the local unit of dermatology and allergy testing.
